# “Negotiating, Navigating, and Networking”: Three Strategies Used by Nursing Leaders to Shape the Adoption and Incorporation of Simulation into Nursing Curricula—A Grounded Theory Study

**DOI:** 10.1155/2014/854785

**Published:** 2014-04-08

**Authors:** Karyn Taplay, Susan M. Jack, Pamela Baxter, Kevin Eva, Lynn Martin

**Affiliations:** ^1^Department of Nursing, Faculty of Applied Health Sciences, Brock University, 500 Glenridge Drive, St. Catharines, ON, Canada L2S 3A1; ^2^School of Nursing, Faculty of Health Sciences, McMaster University, 1280 Main Street West, Hamilton, ON, Canada L8S 4K1; ^3^Centre for Health Education Scholarship, Faculty of Medicine, University of British Columbia, 950 West 10th Avenue, Vancouver, BC, Canada V5Z 1M9

## Abstract

*Background*. Implementing simulation requires a substantial commitment of human and financial resources. Despite this, little is known about the strategies used by academic nursing leaders to facilitate the implementation of a simulation program in nursing curricula. *Methods*. A constructivist grounded theory study was conducted within 13 nursing programs in Ontario, Canada. Perspectives of key stakeholders (*n* = 27) including nursing administrators (*n* = 6), simulation leaders (*n* = 9), and nursing faculty (*n* = 12) were analyzed using the constant comparison method. *Results*. Nursing leaders, specifically nursing administrators and simulation leaders who successfully led the adoption and incorporation of simulation into nursing curricula, worked together and utilized negotiating, navigating, and networking strategies that impacted the adoption and incorporation of simulation into nursing curricula. *Conclusions*. Strategies that were found to be useful when planning and executing the adoption and incorporation of an innovation, specifically simulation, into nursing curricula provide practical approaches that may be helpful to nurse leaders when embarking upon an organizational change.

## 1. Introduction 

The use of simulation as a teaching strategy in nursing education has developed significantly within the past decade [1–3]. Despite the increased use of simulation and the attention received [3], the integration of simulation into nursing curricula has been inconsistent. In 2004/05, the Ontario Government provided each nursing program in the province with approximately $500,000 in funding to purchase simulation equipment [4]. Prior to this time, the use of mid- to high-fidelity simulation equipment as a teaching strategy was uncommon in most programs of nursing. Mid- to high-fidelity equipment is defined as life-like equipment that can imitate real-life responses to medical conditions [5, 6]. What followed was a time of dynamic change in nursing curricula as nursing programs started the process of incorporating simulation which provided an opportune time to examine how organizational culture shapes the adoption and incorporation of simulation.

Taplay and colleagues [7] discovered key organizational elements that shape a common process of adoption and incorporation of simulation into nursing curricula. Institutions that were able to navigate this process and integrate simulation into all levels of curricula in which nursing content was taught were classified as high uptake. The key organizational factor that was identified in high uptake sites was the shared leadership among nursing leaders. This paper represents an effort to delve more deeply into the shared leadership among nursing administrators and simulation leaders and to explain the three leadership strategies (negotiating, navigating, and networking) that played a key role in the adoption and incorporation of simulation into nursing curricula in Ontario, Canada. While the focus of this study was mid- to high-level fidelity equipment, the common term “simulation” will be used throughout this paper.

Leaders engage in processes that bring value to an organization by influencing change [8] thereby shaping organizational culture [9, 10]. Now more than ever, academic nursing leaders are expected to be innovative and facilitate change because nursing education is undergoing a period of great change with the incorporation of new technologies, including simulation. Young and colleagues [11] conducted a phenomenology study exploring the experiences of becoming a nurse faculty leader among a group of 21 nurse educators. The participants in this study often reported that they felt unprepared to assume leadership roles and lacked the skills or strategies needed to manage change. Horton-Deutsch and colleagues [12] identified three strategies used by nurse educators when faced with leadership challenges: “reflecting,” “persevering through difficulties,” and “learning to relate to others in new ways” (page 487). Pearsall and colleagues [13] suggest an additional strategy of “doing your homework” (page 1) as a way to manage change. The researchers found that learning about a subject and weighing the positives and negatives before making decisions lessened their concerns about change when taking risks. They suggested that risk taking is a key factor in academic leadership since it involves trying something different or innovative. Although these researchers identified general strategies used by nursing leaders when they were met with challenges, there remains a gap in the literature related to strategies that academic nurse leaders use when trying to adopt and incorporate new technology. Further insight is needed to understand the processes and strategies nurse leaders use to facilitate the integration of simulation into nursing programs.

## 2. Method 

### 2.1. Design

The principles of grounded theory [14] guided all methodological decisions related to sampling, data collection, and analysis. This approach was used to guide this research because it provided an opportunity to examine how nursing leaders managed the complex process of adopting and incorporating simulation into nursing curricula. A review of institutional mission and vision statements served to provide organizational context and insight into the cultures in which this simulation initiative was occurring [10, 14].

### 2.2. Sampling

Participants from 13 of 34 provincial nursing programs were included in this study. Participants included nursing administrators, simulation leaders, and nursing faculty members. Maximum variation, a method of purposeful sampling, was used to capture the differences in nursing programs by geography and structure of program [14]. All geographic regions of the province were represented as were both college and university nursing programs and the collaborations between them. In addition, maximum variation sampling was used to enhance the degree of representation among the participants themselves.

Theoretical sampling, a hallmark of grounded theory research, helps to explore, define, and recognize attributes of themes as they emerged. This type of sampling continued until no new properties emerged which indicated that theoretical sufficiency was achieved [15].

### 2.3. Data Collection

Data were collected using two rounds of audio-recorded face to face or telephone semistructured interviews. Initial interviews focused on the process of adoption and incorporation of simulation and were approximately 60–75 minutes. Second interviews, focused on emerging categories, in particular the leadership roles which facilitated the process, were approximately 30–60 minutes. NVivo 9 software [16] was used to organize and manage all data.

### 2.4. Data Analysis

All interviews were transcribed verbatim and then analyzed line-by-line and incident-by-incident by the primary investigator (KT). The ensuing codes were developed and defined through the use of the constant comparative method of analysis comparing data within and across sites [14]. The codes were then condensed into categories. Concurrent data collection and analysis, a feature of grounded theory research, was used to aid in the process of developing the categories and in defining the properties and characteristics which led to the nascent structure of the developing theory. To stay true to the participants' perspectives,* in vivo* codes were used and “will be highlighted in quotations throughout the paper”.

### 2.5. Ethics

Two research ethics boards approved this study. Consent was obtained from all participants who were informed that their participation was voluntary. Anonymity and confidentiality were maintained by removing all identifiers and numerically coding the data.

## 3. Results 

### 3.1. Demographic Data

Participants included simulation leaders (*n* = 9), nursing administrators (*n* = 6), and nursing faculty (*n* = 12). All participants were female and registered nurses. All had a baccalaureate degree with the majority having a master's degree (85.1%); 14.8% had a PhD. They ranged in age from 20 to 70 years. The majority (75%) were between the ages of 41 and 60 years. The primary place of employment was almost evenly divided between universities (55.5%) and colleges (44.4%). Participants (37.5%) had an average of 3–5 years (range 1–20 plus years) of experience using mid- to high-fidelity simulators.

Among the sites in this study there was variability in the uptake of simulation ranging from high to low. The cause can be understood in part by considering the leadership differences which presented in this study. The most apparent difference between the high uptake sites and the mid and low uptake sites was the consistent leadership shared between the nursing administrators and the simulation leaders. This shared leadership was the key element that shaped the adoption and incorporation of simulation into nursing curricula.

### 3.2. Nursing Leaders

Nursing administrators were identified as a chair, dean, or director of nursing within their respective nursing programs. In their administrative roles, they were instrumental in the development of the new simulation leader role. Nursing administrators in the high uptake sites realized that the workload would need to be shared and had the insight to create a new role to facilitate the integration of simulation. They also recognized that the simulation initiative required an individual who was willing to take the lead. This was highlighted by one simulation leader who stated:

*The Dean approached me to spearhead the (simulation) initiative and … plan for the acquisition of equipment and facilities. It was considered a special project that I was asked to lead … my job description was altered to accommodate additional responsibilities (002).*

This represented the significant level of responsibility and decision-making power bestowed upon the simulation leaders.

This change in work responsibilities also came with a change in title. The people who took on the role of simulation leader were identified by such titles as simulationists, simulation champions, simulation specialists, or simulation coordinators. The simulation leader role differed considerably across organizations with respect to title, responsibility, and expectations. However, despite the differences, most identified having some if not all of the following responsibilities: developing and sharing expertise about all aspects of simulation; developing or designing simulations; supporting nursing faculty members and clinical instructors in the development of the knowledge and skills to enable their understanding of the equipment's capacity and utilization; providing technological support; managing the facilities; and organizing simulation experiences for students. Some simulation leaders also had the responsibilities of managing simulation committees, creating simulation templates, and motivating people to incorporate simulation into the curriculum.

The diversity of role expectations and responsibilities among the simulation leaders was institutionally driven and based on what worked best at the time for the institution and the nursing program, that is, what was the most feasible and what was the most expeditious to implement. This was highlighted by two simulation leaders describing their role. The first stated: “what I do is design and write out the scenarios and facilitate every simulation that happens in the lab and … get more faculty and staff trained to feel comfortable doing (simulation)” (007). The second stated that her role consists of “primarily overseeing the simulation activities, the physical space, and the logistics of it. Not so much creating the actual simulation or the learning plan objectives, but taking the faculty's vision and bringing it to life” (001). The diversity within this role was further emphasized by organizational classification; some simulation leaders were classified as nursing faculty while others were classified as staff. The inconsistencies in title, responsibility, and work expectations among simulation leaders highlight the challenges associated with a newly developing role.

In the high uptake sites where nursing administrators and simulation leaders shared power, decision-making, and responsibilities related to the integration of simulation, three key strategies emerged that nursing leaders engaged in to facilitate the adoption and incorporation of simulation into their nursing curricula. These included “negotiating,” “navigating,” and “networking” that both nursing administrators and simulation leaders employed either jointly or independently.

### 3.3. Negotiating

The negotiations that leaders engaged in when developing goals and action plans included coming to an agreement regarding the resources and personnel needed to incorporate simulation into the curriculum. While the nursing administrators and simulation leaders were both required to negotiate with individuals, the process started with the administrators. Nursing administrators were involved in negotiations with upper level administration within the institution where they emphasized the importance of the simulation initiative and created awareness of what would be needed to be in place to support this initiative. This was an essential first step, since resources, space, and support from the institution were required to develop the simulation labs, particularly because the funding received from the province was earmarked for the purchase of simulation equipment only. The second step was to convey the need for a lead simulation person. Nursing administrators, particularly those from the high uptake sites, used three strategies during these initial negotiations: education to heighten awareness about the needs of the nursing department, followed by persistence and persuasion. One nursing administrator provided an example of how she had articulated the needs of the nursing program by stating that she had to “educate the Dean about what a nursing lab is, and introduce (simulation) into a culture where there's absolutely no knowledge of it” (005). Another administrator discussed the persistence she used to secure resources by stating “it took a lot of dialogue with senior administration, negotiations around space and the proposals for a simulation coordinator … we were kind of persistent in making the argument” (025). She stated that she approached these negotiations with the philosophy “that you cannot get what you require unless you communicate your needs” (025).

The final negotiating strategy used was persuasion. Persuasion in this context involved emphasizing the institutional benefits that could result from the nursing program adopting and incorporating simulation into the curricula. Nursing administrators typically highlighted three institutional benefits when negotiating with upper level administration. First, integrating simulation was a way to become, or stay, competitive with other nursing programs. Second, having simulation integrated into the curricula could aid in the recruitment of potential students. Third, the accomplishments related to simulation (e.g., securing grants, conducting research, or the lab itself) could be used to publicly promote the nursing program and, in turn, promote the institution as a whole. These strategies used by nursing administrators helped to acquire the necessary resources and personnel for simulation.

Once resources were allocated for space and a new position was created, the nursing administrators were then able to share the negotiating responsibilities with the simulation leaders. The focus turned to increasing buy-in and the use of simulation among nursing faculty members which required different negotiating tactics by the nursing administrator and simulation leader. Nursing administrators created opportunities for faculty to learn about the potential for simulation and encouraged them to consider where simulation fits into courses or curricula. This was done by sharing information at meetings or by supporting the faculty members' attendance at conferences, whereas simulation leaders provided opportunities for nursing faculty members to gain hands-on experience with the equipment thereby enhancing their comfort level and providing opportunities to offer suggestions on how simulation could be incorporated into their specific courses. The institutions took a tandem approach to negotiations. Both types of leaders interacted with faculty members but used different negotiating strategies to implement simulation into the nursing curriculum.

### 3.4. Navigating

Navigating requires finding a way, creating a path, or setting a specific course of action through uncharted territory. It often involves using specific instruments or means. It requires direction or a plan and can be challenging [17, 18]. Participants in this study identified two strategies used to direct the pathway for simulation to be integrated into the curriculum. The first was the leadership style(s) used by the nursing administrators during the adoption and initial incorporation of simulation into the curriculum. The second was the development of the simulation leader's role.

Participants discussed three unique leadership styles employed by nursing administrators when navigating through the adoption and incorporation of simulation into the curriculum: (a) “participatory,” (b) “delegative,” and (c) “laissez-faire.” The first two leadership styles were found in the high uptake sites and were accompanied by a vision or an idea of how an innovation could fit within the current curriculum consistent with charting a path or a course of action when navigating. Participatory leadership encouraged input from all members of the nursing department about the uses for simulation. Leaders who used this strategy presented simulation as a solution that could address challenges with gaps in the curriculum or augment clinical experiences offered to students. This type of leadership encouraged shared decision-making within the nursing department and provided the opportunity for all to have a voice and contribute to the initial and ongoing vision. Sites that used this shared or team approach initially continued to do so as simulation was further integrated into the curriculum.

Delegative leadership primarily involved unilateral decision-making by nursing administrators at the onset of the initiative. Simulation was presented to simulation leaders and nursing faculty members as an expectation by these leaders. Leaders who used a delegative style did not include much if any input from the simulation leader or faculty members into the overall development of a vision. However, once the expectations of the nursing administrators were made clear, the simulation leaders were given power, permission, and domain over how to incorporate simulation. One example of this was stated by a simulation leader:

*Our Dean would tell us … simulation is a priority. Here are my expectations, we need to do this to enhance our curriculum and the way that we get there is totally up to you but here are my expectations (018).*

Nursing administrators who used both participatory and delegative leadership styles were able to encourage both simulation leaders and nursing faculty members to work together which resulted in a higher level of uptake than institutions where the participants reported that a laissez-faire leadership style was employed. These sites had difficulty because they did not or could not establish or convey a plan for simulation or a direction to follow. One faculty member highlighted this by stating that “the director at the time said basically … if you think there is a place for (simulation) to be integrated, find a place” (027).

The second strategy articulated by participants that served to maintain the direction of integrating simulation into the curriculum was the development of the simulation leader's role. In most high uptake sites, simulation leaders were given a new title, power, and autonomy with their new role. This helped nursing programs to navigate the uncharted path of integrating simulation since many simulation leaders invested considerable personal time and effort to develop expertise in this area. To do this, many simulation leaders worked toward creating a new work identity and aligning simulation with their career and educational goals. This was noted by a simulation leader who stated:

*As far as the simulation piece, it just seemed to be a fit … it fell in line with what my organization needed but it also fell into line because I could focus my Masters on (simulation) in nursing education (021).*

While there was substantial personal sacrifice noted, in some cases this resulted in professional achievements such as advancing from part-time to full-time employment status. Gaining expertise provided simulation leaders a means of managing challenges which arose during the integration of simulation such as resistance or indifference among faculty members or troubleshooting equipment problems. The development of the simulation leader's role served to facilitate and direct the path of simulation into the curriculum.

### 3.5. Networking

Networking involves creating or seeking out a support system comprised of individuals or groups who have the same or similar interests and objectives [17, 18]. Participants in this study described networking as the creation of relationships by both the nursing administrator and simulation leader who served to move simulation forward in the nursing curriculum. These connections occurred within the institution, among different professions, outside of the institution, and across the nursing profession. Both nursing administrators and simulation leaders created support systems to gain information and share resources related to simulation. Nursing administrators primarily used networking as a means of collaborating and securing necessary or additional resources, whereas simulation leaders used it for the purpose of learning and gaining expertise.

Within the individual institutions, some nursing leaders connected with other departments representing different disciplines that included physical or occupational therapy, medicine, pharmacology, and emergency response. This strategy allowed the programs to share resources such as lab space, equipment, and, at times, personnel, which provided the potential for institutional cost-savings. Networking with colleagues from other professions within the same organization who have experience or expertise with simulation enabled simulation leaders to learn about the equipment and gain expertise in managing and organizing a lab. Additionally, this networking provided the opportunity for faculty members and simulation leaders from multiple programs to work together, conduct research, and plan and implement simulations. In some instances, these connections also led to the development of interprofessional simulations that met the needs of students in different programs.

Networking also occurred with local health care agencies such as hospitals and community health care organizations. In some cases, the nursing program would reach out to the health care agency to inform them of the educational approaches offered to nursing students through simulation. Other institutions presented simulation to local health care agencies as a means of generating potential revenue by having agencies rent out the facilities and equipment for staff training purposes. Other programs initiated these partnerships as a way to enhance interprofessional education. One administrator summed this up stating: “I felt that (simulation) was an interprofessional initiative for the whole region, that simulation would be a way to bring everybody together and raise the profile of this school” (005).

Simulation leaders also connected with other nursing professionals. These connections typically developed through simulation conferences. At the onset of this initiative, most networking was done outside the province, in the United States, since there were few nursing experts in Ontario with whom to consult. These interactions provided an opportunity to learn about nursing-specific content and for simulation leaders to develop their own expertise. Institutions that were able to support simulation leaders' attendance at conferences and thus gain expertise had an easier time integrating simulation into the curriculum.

Networking with the purpose of securing resources that benefited more than one program within the same institution and connecting with health professionals in the community both served to move simulation forward to become a faculty-wide or community affiliated initiative rather than just a nursing-specific initiative. Networking was a key strategy used by nursing leaders during the preliminary phases of the simulation initiative, but it must continue in order to advance simulation in nursing education. It needs to be actively pursued by both nursing administrators and simulation leaders.

## 4. Discussion 

The tandem leadership between the nursing administrator and the simulation leader is similar to the definition of shared leadership that exists in the literature. Shared leadership is considered a dynamic interaction between people that focuses on achieving specific group or organizational goals [19]. While this type of leadership is discussed within the broader field of education [20], there is no discussion of how it has been applied to simulation. This is a significant finding from this study that adds to the literature on simulation. To date, much of the literature about the uptake of simulation into nursing curricula has focused on the attitudes and beliefs of faculty members [21] and the aspects that nursing faculty consider when making decisions about whether to incorporate simulation [22]. This study, on the other hand, suggests that faculty attitudes and beliefs about simulation as a teaching strategy may not be the only consideration which can facilitate or impede the adoption and incorporation of simulation. The shared leadership between the nursing administrator and the simulation leader who utilize negotiating, navigating, and networking strategies to manage change contributed significantly in the adoption and incorporation of simulation into nursing programs. Sites that had leaders working in tandem to share the workload and the responsibilities experienced a high level of uptake of simulation compared to sites that did not have these nursing leaders. Effective shared leadership involved utilization of negotiating, navigating, and networking strategies to manage change.

The role of simulation leader proved to be crucial in the process of adopting and incorporating simulation into nursing programs. The findings from this study provide insights into the complexity and diversity of this role by highlighting the multiple responsibilities and extensive workload expectations. What was discovered during this study is that the development of the role of the simulation leader was driven by the needs, requirements, and feasibility of each individual institution. This was highlighted by the lack of consistency related to workload, level of responsibility, and title. As a result, the role may become indistinguishable from the institution because it is so specifically based on the needs and resources within that organization. This can potentially lead to ambiguity about the role of simulation leaders as it relates to the broader context of the nursing culture. This is an issue for future consideration as the role of the simulation leaders becomes embedded into the organizational structure of nursing programs.

## 5. Strengths and Limitations

The strengths of this study included the triangulation of data sources and theoretical sampling. Triangulation of data sources was achieved by including participants who held different roles in the same institution, thus offering varied perspectives [23]. Theoretical sampling was achieved by returning to participants to clarify concepts and add further details in order to refine the emerging theoretical categories [14, 24]. This strategy was used “until no new properties emerge[d]” [14, page 96].

A limitation of this study was that the chair, dean, or the director roles within the nursing programs were grouped together under the umbrella of nursing administrator. The roles were not differentiated with respect to specific responsibilities or the permanence of the position. Inclusion of these aspects may have elicited additional findings related to the shared leadership among the nursing leaders.

## 6. Conclusion

Nursing leaders, specifically nursing administrators and simulation leaders who represented high uptake sites, worked in tandem and utilized negotiating, navigating, and networking strategies to impact the uptake of simulation into nursing curricula. Nursing leaders who employed these strategies were able to secure necessary resources, collaborate with key stakeholders, gain information, create a vision, and forge a course of action through uncharted territory. Insights regarding the development of the role of the simulation leader were shared and concerns about the future of this role as it relates to the broader context of the nursing profession were raised. Additionally, this study offered strategies that may be useful when planning and executing the adoption and incorporation of an innovation, specifically simulation, and offered practical approaches that may be helpful to nurse leaders when embarking upon an organizational change.
